# Stem cell therapy combined with controlled release of growth factors for the treatment of sphincter dysfunction

**DOI:** 10.1186/s13578-023-01009-3

**Published:** 2023-03-16

**Authors:** Shengzhou Shan, Qingfeng Li, Tracy Criswell, Anthony Atala, Yuanyuan Zhang

**Affiliations:** 1 Department of Plastic and Reconstructive Surgery, Shanghai Ninth People’s Hospital, Shanghai Jiao Tong University School of Medicine, Shanghai, 200011 China; 2 Wake Forest Institute for Regenerative Medicine, Wake Forest School of Medicine, Winston-Salem, NC 27157 USA

**Keywords:** Sphincter dysfunction, Incontinence, Cell-based therapy, Growth factors, Controlled release system, Neuromuscular regeneration

## Abstract

Sphincter dysfunction often occurs at the end of tubule organs such as the urethra, anus, or gastroesophageal sphincters. It is the primary consequence of neuromuscular impairment caused by trauma, inflammation, and aging. Despite intensive efforts to recover sphincter function, pharmacological treatments have not achieved significant improvement. Cell- or growth factor-based therapy is a promising approach for neuromuscular regeneration and the recovery of sphincter function. However, a decrease in cell retention and viability, or the short half-life and rapid degradation of growth factors after implantation, remain obstacles to the translation of these therapies to the clinic. Natural biomaterials provide unique tools for controlled growth factor delivery, which leads to better outcomes for sphincter function recovery in vivo when stem cells and growth factors are co-administrated, in comparison to the delivery of single therapies. In this review, we discuss the role of stem cells combined with the controlled release of growth factors, the methods used for delivery, their potential therapeutic role in neuromuscular repair, and the outcomes of preclinical studies using combination therapy, with the hope of providing new therapeutic strategies to treat incontinence or sphincter dysfunction of the urethra, anus, or gastroesophageal tissues, respectively.

## Introduction

### Backgrounds of sphincter dysfunction

**Fig. 1:**
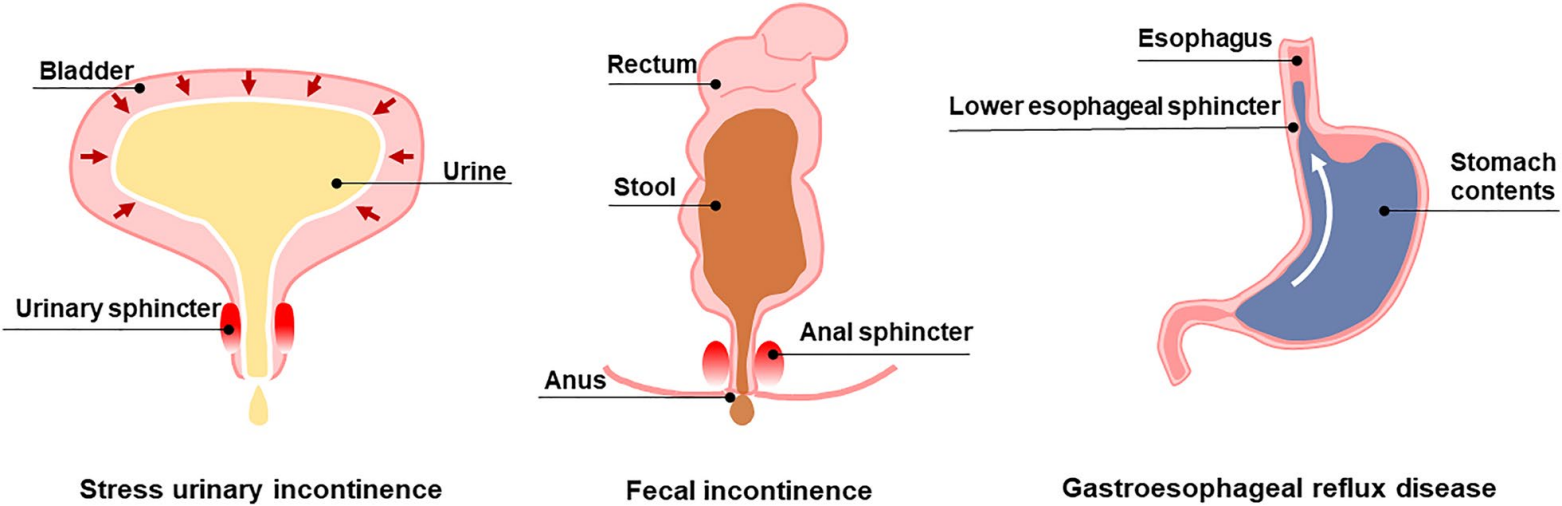
Schematic illustrations of three sphincter dysfunctions. Dysfunction in the urinary sphincter, the anal sphincter, and the lower esophageal sphincter

Sphincters are unique circular muscles that control the passage of fluids or semi-fluids from one tissue to another. The intersection of the two tissues where the sphincter resides is richly innervated with α-adrenergic receptors which are the primary mechanism of continence control. The action of the sphincters is primarily involuntary and controlled by the autonomic nervous system, although there is modest voluntary control through the somatic nervous system. Despite the presence of more than ten types of sphincters in the body, dysfunctions in three of them, located at the proximal urethra or the bladder neck (urinary sphincter), the anus (anal sphincter), and the gastroesophageal junction (lower esophageal sphincter, LES), result in the majority of sphincter-related pathologies. The decrease or loss of sphincter muscle tone due to aging, trauma, or inflammation results in symptoms such as stress urinary incontinence (SUI), fecal incontinence (FI), or food reflux (gastroesophageal reflux disease, GERD), which significantly affect the quality of life of millions of people worldwide (Fig. 1). It is reported that SUI affects about 50% of the female population over 45 years old and about 20% of men over 70 years old [1]. International population-based studies indicated the prevalence of FI is 0.4 to 18% [2]. The burden of GERD continued to worsen with the prevalent cases increasing by 77.53% from 1990 to 2019 [3]. Despite the success of pharmacotherapy and surgical treatment, these approaches provide symptomatic relief only and fail over time since they do not address the underlying cause of the dysfunction- neuromuscular damage. Table 1 summarizes the similarities and differences in dysfunction that occurs in the three types of sphincters. These 3 sphincter dysfunctions will be the primary focus of this review.

**Table 1:** Overview of similarity and difference in three sphincter dysfunctions

Terminology	Stress urinary incontinence	Fecal incontinence	Gastroesophageal reflux disease
Incidence rate	50% (F, ≥ 45 yrs) 20% (M, ≥ 70 yrs)	11 to 15%	18 to 28%
Structure	Urinary sphincter	Anal sphincter	LES
Location	Between ladder and urethra	Anorectum	Gastroesophageal junction
Causes	Vaginal childbirth injury and aging in women; neuromuscular injury after radical prostatectomy in men	Obstetrical surgical injury	Obesity
Mechanistic Effect	Urethral sphincter impairment or weakness due to pelvic floor muscles and nerve injure	Anal sphincter impairment or weakness	Low LES pressure, transient LES relaxation
Function testing	UPP, LPP, RUPP, EMG, ENG, bladder capacity, contractility test	EMG, anal pressure, contractility test	LES pressure measurement, pH monitoring
Histological evaluation	H&E, Masson trichrome for college compound, picrosirius red for collagen networks, Hart elastin for elastin content, Gordon and Sweet staining for reticular (retic) fibers Immunohistochemical stain: Muscle*: desmin, myosin, α-SMA, myogenin, Myo D, Myf-5;* Peripheral nerve: *neurofilament, PGP9.5, βII-tubulin, S100;* NMJ: *neurofilament, α-Bungarotoxin, phalloidin*		
Symptoms	Urine leakage when intra- abdominal pressure increases (e.g., coughing)	Stool leakage	Frequent reflux of gastric contents into the esophagus causing heartburn, regurgitation, and esophageal chest pain
Complications	Emotional and social distress, skin irritation, and mixed urinary incontinence	Emotional and social distress and skin irritation	Esophageal stricture, Barrett’s esophagus, and esophageal adenocarcinoma
Nonsurgical therapy Surgical therapy	Pelvic floor exercises, behavioral modification	Pelvic floor exercises, biofeedback	behavioral modifications, (e.g., lose weight), PPIs
	SLING procedure or injectable bulking agents	Sphincteroplasty or injectable bulking agents	Fundoplication surgery

*EMG* electromyography, *ENG* electroneurography, *LES* lower esophageal sphincter, *UPP* urethral pressure profile, *LPP* leak point pressure, *NMJ* neuromuscular junction, *PGP9.5* protein gene product 9.5, *PPIs* proton pump inhibitors, *RUPP* retrograde urethral perfusion pressure

### The pathogenesis of sphincter dysfunction

**Fig. 2:**
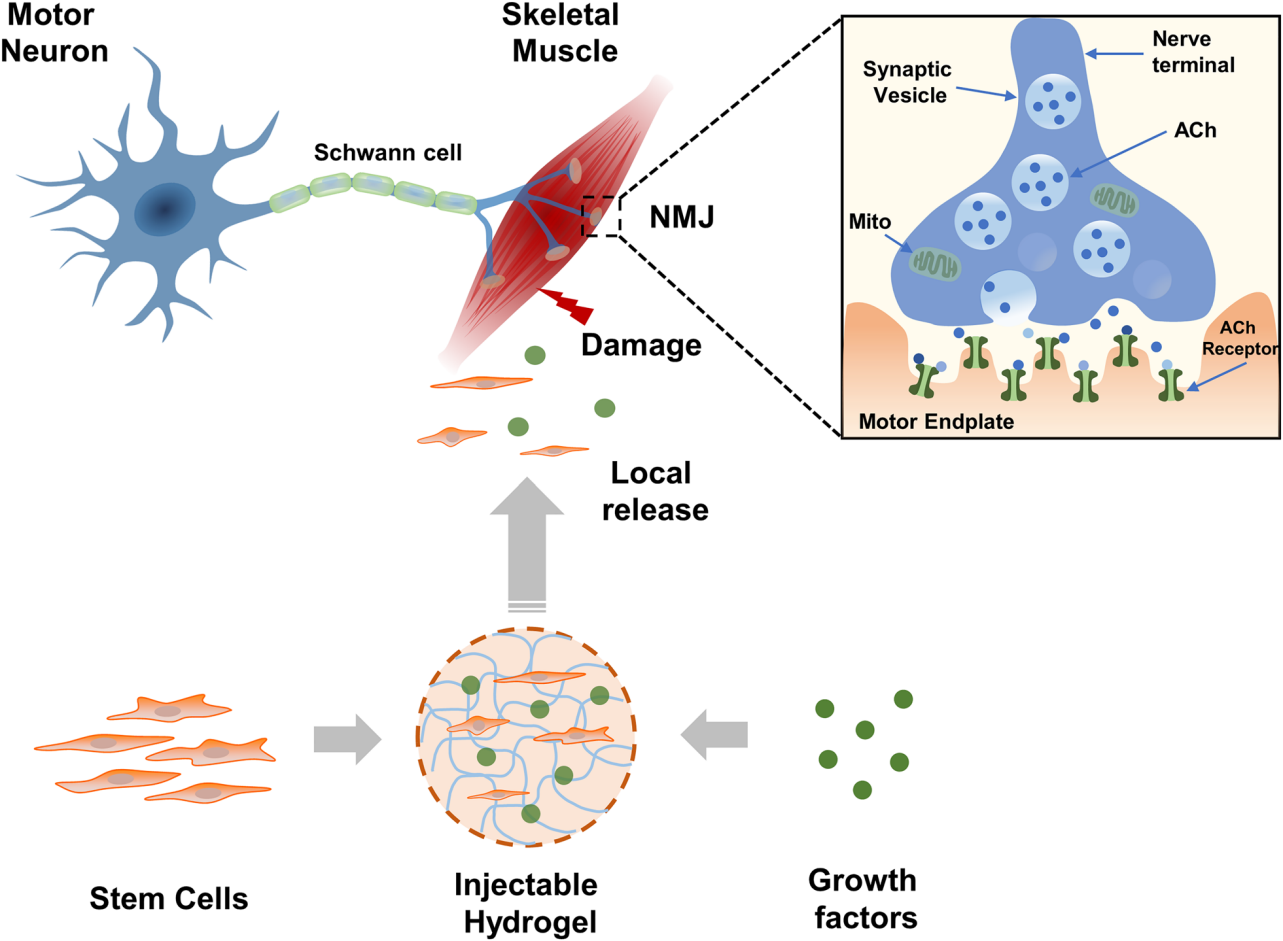
Schematic representation of a biomaterials-based delivery system. The motor neuron, skeletal muscle, and neuromuscular junction (NMJ) contribute to a complete neuromuscular functional unit. The motor neuron axon branches as it comes into contact with the skeletal muscle to form a single NMJ with each individual muscle fiber. At the motor neuron terminal, Ach is released from the synaptic vesicles into the synaptic cleft where it binds to its receptors in the motor endplate to trigger muscle contraction. Injectable hydrogels with encapsulated stem cells and/or growth factors can be used to achieve controlled release into the area of tissue damage. ACh, acetylcholine; Mito, mitochondrion

Muscle weakness as a result of neuromuscular damage primarily originates from three sources: [1] Neuropathy, or damage to the peripheral nerves, caused by diabetes, trauma, certain medications or toxins, alcoholism, inherited factors, etc.; [2] Myopathy, or dysfunction of muscle, caused by mitochondrial defects, certain medications or toxins, inflammation, infection, and metabolic or endocrine problems; and [3] Dysfunction of the neuromuscular junction (NMJ) at the interface of the nerve and muscle as a result of toxin exposures and inherited factors. The NMJ is a highly specialized chemical synapse, capped by terminal Schwann cells and kranocytes, through which a motor neuron interacts with a muscle fiber. When an action potential reaches the synaptic cleft of the motor neuron, the neurotransmitter acetylcholine (ACh) is released from synaptic vesicles, which bind to and activate the ACh receptors on the muscle fiber. Binding of Ach to its receptor results in depolarization of the muscle fiber which triggers calcium release to induce contraction (Fig. 2) [4, 5]. A complex network of effectors and signaling pathways have been implicated in regulating NMJ development and maintenance [5–9]. Due to the essential role of NMJ in the excitation-contraction coupling of the neuromuscular response, the impairment of NMJ structure and function are hallmarks of various neuromuscular disorders including amyotrophic lateral sclerosis (ALS), muscular dystrophies (Duchenne’s muscular dystrophy (DMD) and Becker’s muscular dystrophy (BMD)). Moreover, the NMJ is the site of action for various intoxicating agents, such as botulism.

The pathophysiological process of muscle weakness in sphincter muscle is often multifactorial. For example, vaginal birth injury is the major cause of neuromuscular urinary continence in women leading to the pathogenesis of SUI. It has been demonstrated that vaginal birth injures the external urethral sphincter, the pudendal nerve which innervates the external urethral sphincter, and the intersecting NMJs [10–15]. In addition, several risk factors have been identified such as obesity, increasing age, smoking, diabetes and hormonal changes [1, 16]. For male SUI, surgical treatment of benign prostatic hyperplasia and prostate cancer that impair the innervation or structural components of the internal and external urethral sphincters represent the most common scenarios [17]. At molecular level, many genes are involved in the pathogenesis of SUI which are associated with extracellular matrix metabolism, estrogen receptors, oxidative stress, apoptosis, inflammation, neurodegenerative processes, and muscle cell differentiation and contractility [18]. Similarly, the most common cause of anal sphincter disruption is vaginal birth injury which involve the external anal sphincter, the internal anal sphincter, pudendal nerves, or a combination of these structures [19]. LES is the most important structure at the esophagogastric junction which creates a high-pressure zone that prevents the reflux of gastric contents and bile into the esophagus. An abnormal LES pressure, frequent transient LES relaxation and esophageal clearance impairment are generally thought to be key factors to the pathogenesis of GERD. Factors that decrease LES pressure include hormones (e.g., progesterone in pregnancy), medications (e.g., calcium channel blockers) and specific food (e.g., fat, chocolate) [20, 21]. Taken together, effective restoration of the sphincter muscle and corresponding nerves is essential to achieve a suitable unity of the structure and function of bladder, anus, and the gastroesophageal junction.

### The mechanism of neuromuscular regeneration after damage

Muscle tissue is divided into three basic types: smooth muscle, cardiac muscle and skeletal muscle. Skeletal and cardiac muscles are known as striated muscles [22]. For example, the urethral sphincter consists of the external urethral sphincter and the internal urethral sphincter. The internal urethral sphincter is made up of smooth muscle [23]. The external urethral sphincter is made up of skeletal muscle and is vulnerable to injury during vaginal delivery [10–13, 23]. Skeletal muscle has the ability to regenerate after minor injury [24, 25]. The myogenic regeneration process relies on activation and proliferation of satellite cells, myoblast differentiation and fusion into multinucleated myotubes, and then myotubes hypertrophy and remodeling to generate mature muscle fibers [26]. Injured peripheral nerves also have the capacity to spontaneously regenerate and reinnervate skeletal muscle. After injury, the distal stump of the nerve undergoes Wallerian degeneration. Macrophages are recruited into the injury site to clear the debris. Schwann cells actively proliferate, elongate and align in columns to create a nutrient-rich environment to guide and support the axonal regeneration towards the periphery with a velocity of about 1 mm/day [27–29]. However, the ability of skeletal muscle and peripheral nerve to regenerate is limited, especially when there is a large volume of muscle loss and a lack of guidance for axons across the gap lesion. In addition, the capacity for axonal regeneration depends on the age of the patient, the type of injury and the proximity of the injury to the nerve cell body. In humans, axonal regeneration must occur over much longer distances than in rodents. During long periods without axon regeneration, the target tissues become chronically denervated, resulting in muscle atrophy, as evidenced by a reduced number of muscle fibres [30–32]. Despite significant advances in our understanding of peripheral nerve biology and the mechanisms of nerve regeneration, effective treatments for peripheral nerve injurie remain limited. This is due, in part, to the complex structure of peripheral nerves and the challenges associated with promoting the regrowth and functional reconnection of nerve fibers across the site of injury. Several approaches are being developed and tested to promote peripheral nerve regeneration, including the use of controlled release of neurogenic growth factors, stem cells, and nerve guidance channels [28, 31]. In addition, recent advances in biomaterials and tissue engineering offer new hope for the development of porous scaffolds coated with growth factors that may help promote nerve regeneration and functional recovery after injury [33, 34]. Much more research is needed to fully understand the complex process of peripheral nerve regeneration and to develop effective and reliable treatments for peripheral nerve injury.

**Fig. 3:**
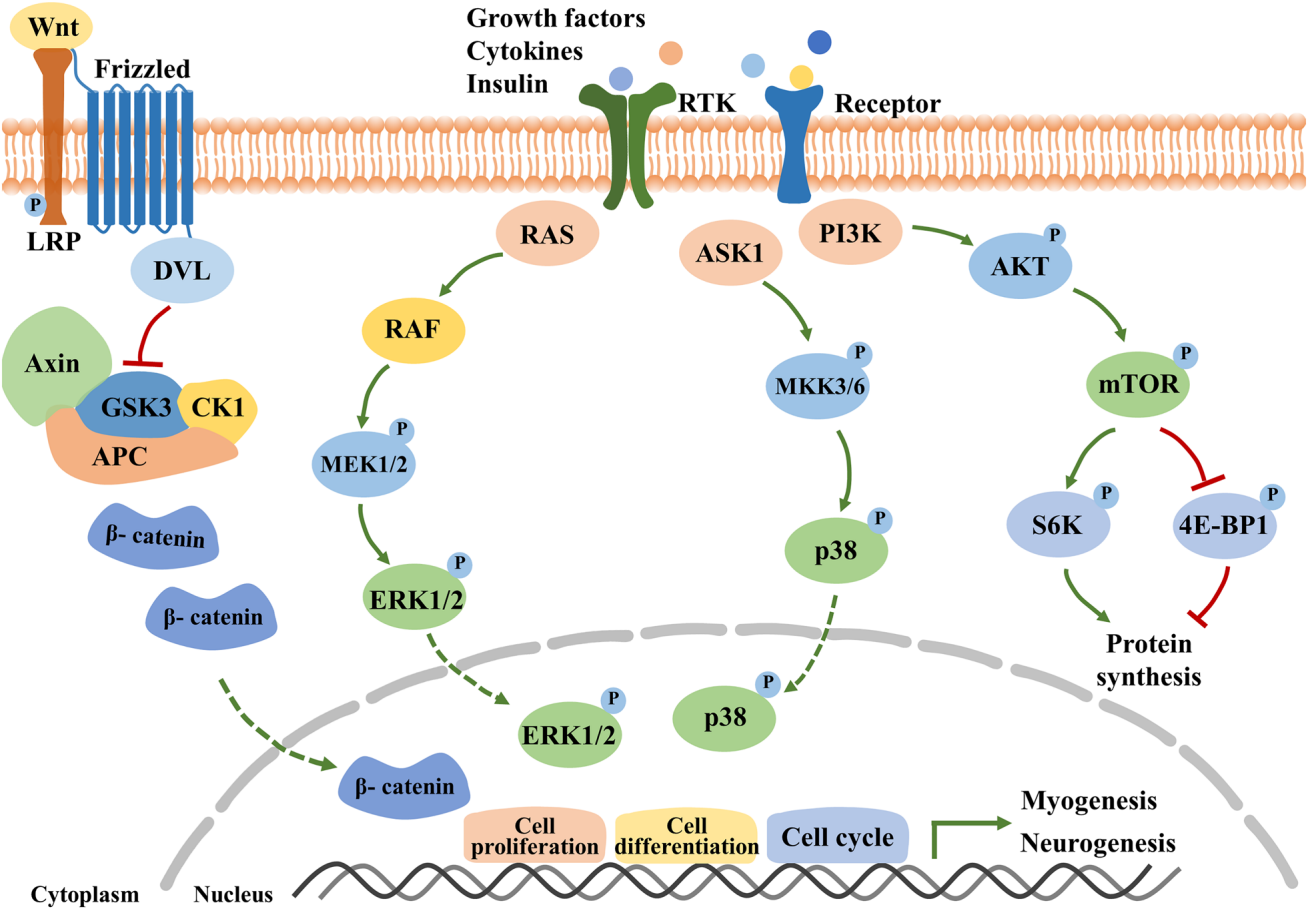
Schematic of the Wnt, MAPK, and PI3K-AKT-mTOR signaling pathways. The Wnt pathway is activated when Wnt proteins bind to Frizzled-LRP receptor complex composed of Frizzled and LRP resulting in translocation of β-catenin to the nucleus, ultimately targeting cell differentiation. The MAPK and PI3K-AKT pathways are initiated by the binding of various small proteins (growth factors, cytokines and insulin) to their respective receptor tyrosine kinase (RTK). The MAPK cascade results in translocation of the Erk1/2 complex to the nucleus and targets cell proliferation pathways, whereas the PI3K-AKT pathway results in mTOR activation and the regulation of protein synthesis. Green arrows indicate positive regulators, whereas red arrows indicate negative regulators

At the molecular and biochemical level, the post-injury neuromuscular regeneration process is regulated by the coordinated activation of various intracellular signaling pathways, such as the Wnt, MAPK and PI3K-Akt-mTOR pathways, all of which are involved in the regulation of cell proliferation, cell differentiation, and protein synthesis during skeletal muscle and peripheral nerve regeneration [35–44] (Fig. 3). In addition, Notch signaling is required to maintain quiescent state for satellite cells by the regulation of self-renewal and differentiation [45, 46] and required for perineurial migration and differentiation during nerve formation, but not regeneration [47]. The JAK-STAT pathway is activated to regulate the myoblasts differentiation positively or negatively [48]. Hippo pathway and YAP/TAZ are indicated to regulate Schwann cell proliferation and differentiation during peripheral nerve regeneration [49]. Thus, the precise orchestration of multiple cytokines and signaling cascades for repair of the different types of damaged tissue (muscle, nerve) is required to achieve full functional recovery of neuromuscular tissue.

### Combination therapy of stem cells and growth factors

Stem cells possess the potential for self-renewal and multilineage differentiation (potency) [50]. Under specific conditions stem cells can be differentiated into multiple types of functional cells and can be used to compensate for lost cells in damaged tissue, including skeletal and smooth muscle, and peripheral nerve tissue repair in the urethral sphincter. Therefore, stem cell transplantation provides a novel and promising therapeutic strategy for neuromuscular tissue regeneration. Most studies have focused on the use of autologous muscle derived stem cells (MDC) to improve urethral sphincter function. Sèbe et al. conducted a prospective study to evaluate the safety and efficacy of intrasphincteric injections of MDC in 12 female SUI patients. The results showed that 3 patients were dry at the 12-month follow-up, 7 others had an improvement in their condition, and 2 patients had a slight worsening of their condition. No serious adverse effects were observed [51]. Another prospective study by Carr et al. found that intrasphincteric injections of MDC improved SUI symptoms at the 12-month follow-up, with better clinical outcomes observed in patients receiving higher doses [52]. Stangel-Wojcikiewicz et al. published a 2-year follow-up study of MDC for SUI. The results showed a 75% success rate, with 50% of subjects cured and 25% of subjects achieving partial improvement [53]. Although encouraging results have been achieved in early clinical trials, wide variations in MDC isolation techniques, the route of administration and cell dosage prevent adequate comparison with MDC transplantation for sphincter dysfunction. Several limitations to the use of stem cells alone include poor cell retention and engraftment in the tissue after transplantation [54]. Implantation of growth factors combined with stem cells has shown potential for remarkably improving stem cell viability, differentiation potential, and function [55–61]. Thus, a combination therapy of stem cells and growth factors may provide optimal therapeutic impact in sphincter tissue regeneration and functional restoration.

**Fig. 4:**
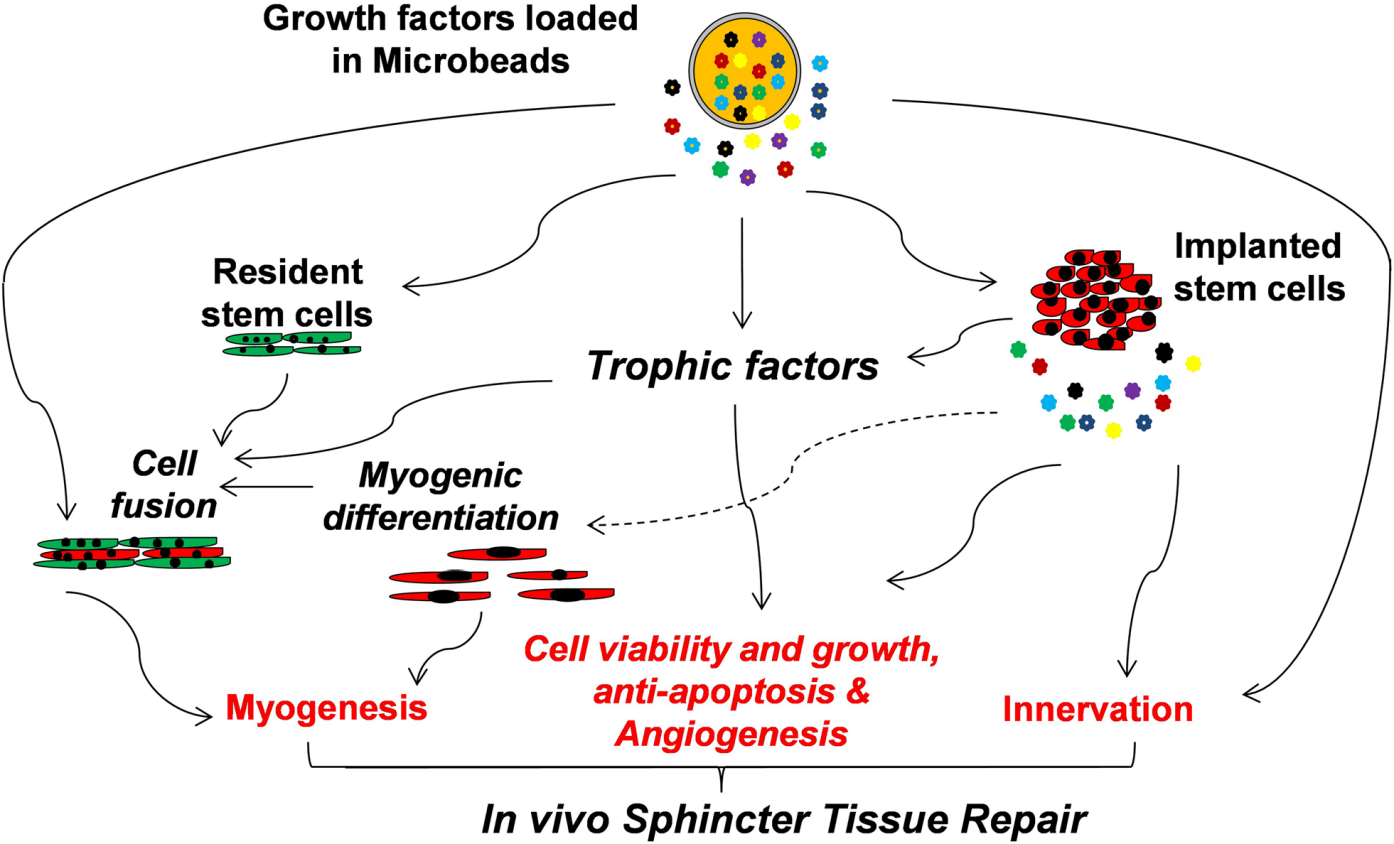
The working model of stem cell-growth factor therapy. A combination therapy of stem cells and controlled release of growth factors enhances cell viability and differentiation potential, stimulates endogenous stem cells and provides an optimal microenvironment for sphincter tissue repair and regeneration

Bioactive growth factors are soluble secreted proteins that participate in stem-cell-based tissue repair and regeneration and enhance stem cell therapeutic efficacy by interacting with cellular specific receptors to initiate extracellular and intracellular signaling cascades responsible for cell physiological function. Concerning stem cell transplantation, many of these growth factors modify the microenvironment of the recipient tissue to support donor cell survival, proliferation, and differentiation. However, the therapeutic use of growth factors is largely limited due to their inherent instability, short half-life, and rapid deactivation in vivo [62, 63]. To achieve a sufficient therapeutic effect, direct injection of a supra-physiological dose of growth factors may be required, which can lead to adverse effects, such as tissue edema, system hypertension, and an increased risk of cancer [64, 65]. An efficient control release system could bypass many of these limitations and result in improved growth factor therapy (Fig. 4).

### Advanced delivery system for dual therapy

To overcome the shortcomings discussed above, genetic approaches and biomaterial-based strategies have been developed to optimize tissue regeneration in response to these therapies. Genetic modifications of cells to overexpress the growth factor of interest prior to implantation has been used to sustain secretion of growth factors into injured tissue post-transplantation [54]. In addition, the use of different biomaterials, conjugated with growth factors, have been shown to support a microenvironment that mimics the ideal native extracellular matrix for a particular cell type.

Injectable hydrogels are good materials for the controlled release of growth factors, as well as a stem cell delivery system, by offering protection against growth factor degradation, a reduction of growth factor dose leading to decreased adverse side effects, and enhanced retention and survival of the encapsulated cells [63, 66, 67] (Fig. 2). Biomaterials can generally be classified into two major types according to their origin: natural materials and synthetic materials. Natural materials are derived from naturally occurring polymers and include proteins (e.g., collagen, gelatin, silk and fibrin), polysaccharides (e.g., alginate, starch, chitosan, hyaluronan, and chondroitin sulphate) and tissue extracellular matrix (e.g., small intestinal submucosa). Synthetic materials, or materials synthesized in a lab, include polyacrylic acid (PAA), poly lactic-co-glycolic acid (PLGA), polycaprolactone (PCL) and polyethyleneglycol (PEG) [68, 69].

Natural materials are highly biocompatibility and easily degrade over time after transplantation. Hydrogels, one of the more commonly used natural materials, swell to form 3D structures that provide an ideal environment to support cell adhesion and tissue formation [70]. However, natural materials, especially hydrogels, have relatively poor mechanical properties and controlling their physicochemical properties is difficult. Synthetic materials have the ability for photopolymerization, they have adjustable mechanical properties, and thus, easily modified architectural and chemical properties; but bioactivity is lacking. To take advantage of the bioactivity of natural scaffolds and the modifying properties of synthetic materials, natural-synthetic polymer hybrids are being studied for tissue engineering purposes [71, 72].

## Stem cell therapy

### Mechanisms of stem cell therapy

The most common types of stem cells that have been used and evaluated in preclinical and clinical studies for neuromuscular tissue repair and regeneration include: [1] pluripotent stem cells (PSC), including embryonic stem cells (ESC) and induced pluripotent stem cells (iPSC); [2] mesenchymal stem cells (MSC), including bone marrow-derived mesenchymal stem cells (BMSC), adipose-derived mesenchymal stem cells (ASC), human umbilical cord mesenchymal stem cells (HUMSC), endometrial mesenchymal stem cells (EMSC), and dental-derived mesenchymal stem cells (DMSC); and [3] adult stem cells including MDC and urine derived stem cells (USC).

Stem cells remain quiescent in normal healthy tissue. After injury, stem cells become activated in response to cytokines and other bioactive molecules secreted by the tissue and immune cells at the site of injury at which point they begin dividing and differentiating to replace the damaged tissue. In animal models of neuromuscular tissue injury, stem cells have been demonstrated to enhance functional recovery after transplantation via a couple of different mechanisms. In addition to differentiation and integration into host tissue, transplanted stem cells can interact with the host tissue without differentiation through intercellular interactions with host cells and microenvironment, and the secretion of paracrine factors including neurotrophic support, immune regulation, and modulation of metabolic signaling [73, 74].

Although studies have shown that stem cell transplantation promotes regeneration of striated and smooth muscle layers, as well as nerve fibres in the sphincter [75–80], the mechanisms of stem cell function differ in each cell type. The regenerative abilities of MSC are mainly mediated by paracrine activity of trophic factors, transfer of mitochondria by tunneling nanotubes, and transfer of molecules from exosomes or microvesicles [81–84]. Of note, the directed differentiation of MSC into damaged tissue after transplantation remains controversial. Some studies have shown that a small number of transplanted MSC differentiate into striated and smooth muscle cells [76, 85]. However, other studies have confirmed that cell fusion rather than MSC transdifferentiation appears to be responsible for tissue regeneration [86]. The regenerative abilities of MDC and USC are mainly attributed to their multilineage differentiation capabilities, which can differentiate not only into myogenic lineage but also into neurogenic lineage [87–93]. In addition, the paracrine effects of USC have been demonstrated, suggesting an immunomodulatory property similar to that of MSC [94, 95]. PSC are theoretically capable of differentiating into any cell type, providing an unlimited source for the generation of myogenic and neural precursors. The iPSC are adult cells reprogrammed by the introduction of specific pluripotency factors. They share many of the characteristics of ESC, while offering many advantages over ESC, including simplified ethical concerns and reduced risk of immune rejection. In addition, they would allow the generation of autologous patient-specific stem cells [96]. Studies have shown that transplantation of human ESC-derived and iPSC-derived smooth muscle progenitor cells facilitates the restoration of smooth muscle tissue and urethral sphincter function [97–99]. Although iPSC and ESC can differentiate into each cell type of the sphincter tissue, including smooth muscle, skeletal muscle cells, peripheral never cells, neuromuscular junction, and endothelial cells [100–105], studies combining all of these cells together to form the sphincter have not been reported yet. This is an exciting area of research with great potential for therapeutic applications using iPSC derived from the patient’s somatic cells, but there are still many technical and scientific challenges that need to be overcome in order to fully realize the potential of iPSC and ESC to regenerate functional sphincter tissue. The main concerns are the safety of the use of iPSC/ESC and the precise connection of the implanted tissue-engineered sphincter tissue to the host tissue via vessel to vessel, nerve to nerve, or muscle to muscle, respectively.

### Pre-clinical data in sphincter dysfunction

In preclinical animal models of sphincter dysfunction, stem cell transplantation has been tested with variable degrees of success. Improved sphincter function including increased leak point pressure, bladder capacity and anal pressure were observed after stem cell application (Table 2). However, most of these studies were focused on the urinary and anal sphincter, partly due to the difficulty of administering stem cells into the LES. In one study, endoscopic injection of skeletal muscle-derived cells into the LES of a canine model reported a significant increase in LES pressure. The transplanted cells were shown to differentiate into mature skeletal muscle in the sphincter [106]. In another study, the investigators observed that transplanted bone marrow-derived cells induced epithelial regeneration in the gastrointestinal tract. Although LES function was not assessed in these experiments, this study provided a proof-of-concept that systematic application of stem cells for the treatment of esophageal ulcers which are mostly caused by GERD [107, 108]. In the selection of stem cell sources, MDC, BMSC, and ASC were the most commonly used in these studies due primarily to their relative ease of harvest. From the perspective of clinical practice, an ideal cell source should be ethically acceptable, non-invasive and inexpensive to collect. As a by-product of human metabolic waste, USC are easily obtained from urine. USC have a high proliferative capacity due to their higher telomerase activity than BMSC. A USC clone can expand to generate a large number of cells, which is a great advantage for cell-based therapy in tissue repair and regeneration [87, 109]. USC have been shown to have the capacity for multipotent differentiation and immunomodulation with strong paracrine effects [87, 94, 110]. It has been reported that USC were able to differentiate into neuronal lineage cells after transplantation into the rat brain [111]. When implanted subcutaneously in nude mice, myogenic differentiated USC can form multiple layers of smooth muscle cells [87]. In a rat model of SUI, USC exosomes were effective in improving the urodynamic parameters and repairing damaged muscle [112]. These encouraging results from animal studies provided a solid basis for further development of USC-based therapy in neuromuscular regeneration. However, the lack of standard procedures for USC isolation and quality control hinders the progress of USC into clinical applications [94]. How to obtain clinical-grade USC, optimal cell dosage and delivery method should be the focus of future research.

**Table 2:** Selected preclinical studies using stem cell transplantation for sphincter dysfunctions

Goal of studies	Animal model	Source and number of stem cells/ transplant methods	Main outcomes	Conclusions
Urethral sphincter				
Evaluated the effects of MDC/BMSC co-transplantation into the urethra for SUI	Goats/ adult/aged, multiparous	29.6 × 106 goat MDC/ BMSC / Intraurethral injection [113]	↑ UPP, ↑ Ske-M	MDC/BMSC co-transplantation provides a greater chance of improvement in urethral closure than transplantation of each population alone
Determined whether allogenic MDC could restore sphincter function	Rats/ cauterized urethra	1.5 × 106 rat MDC/ Intraurethral injection [231]	↑ LPP, ↑ Ske-M, ↑ Peri-N	Injection of intraurethral MDC improved sphincter function in rats with intrinsic sphincter deficiency
Determined if increasing numbers of MSC improves urethral and pudendal nerve function and anatomy	Rats/ PNC + VD	one, two, or three doses of 2 × 106 rat BMSC/ IV [75]	↑ LPP, ↑Ske-M, ↑Sm-M, ↑Peri-N	MSC therapy for postpartum incontinence and SUI can be enhanced with multiple doses
Assessed the effect of BMSC on LPP changes in a rat model of SUI	Rats/ PNC	1 × 106 rat BMSC/ Periurethral injections [114]	↑ LPP, ↑Ske-M	Periurethral injection of MSC in an animal model of SUI restored the damaged external urethral sphincter
Evaluated functional and histological recovery by autologous BMSC transplantation into injured urethral sphincters	Rats/ urethral sphincters injury by urethrolysis and cardiotoxin injection	2.0–5.0 × 105 rat BMSC/ Periurethral injections [76]	↑Bladder capacity, ↑ Ske-M, ↑ Peri-N, but no changes in LPP	A trend towards recovery of LPP in BMSC-transplanted urethras
Investigated the injected autologous ASC in improving SUI	Rats/ VD	2.0–5.0 × 105 rat ASC/ Periurethral injections [77]	↑ LPP, ↑ Bladder capacity, ↑Ske-M, ↑Sm-M, ↑ Vas	Periurethral injection of ASC improved urinary function and urethral structure in SUI rats
Investigated whether transplantation of ASC can treat SUI	Rats/ VD	1 × 106 rat ASC/ Urethral injections or IV [232]	↑ LPP, ↑ Sm-M	Transplantation of ASC was effective in the treatment and/or prevention of SUI
Anal sphincter				
Determined whether the injection of MDC into the anal sphincter can improve functional properties in a fecal incontinence model	Rats/ cryoinjured anal sphincter	3 × 106 rat MDC/ IM [79]	↑Ske-M, ↑Sm-M	Implanted autologous MDC improved anal sphincter function
Investigated the ability of injected BMSC to enhance sphincter healing after injury and primary repair in vivo	Rats/ SP	0.75 × 106 rat BMSC/ IM [233]	↑Ske-M, ↑Sm-M, ↑contract	BMSC injection improved muscle regeneration and increased contractile function of anal sphincters
Investigated the ability of rabbit BMSC/ human UCMSC to improve anal sphincter incontinence	Rabbits/ SP	104 rabbit BMSC/ human UCMSC/ IM [78]	↑ Ske-M, ↑contract	Stem cell injection at the site of injury enhanced contractile function of the anal sphincter without surgical repair
Estimated the effect of myogenic stem cells on contractile function of the external anal sphincter after transection	Rats/ SP	3.2 × 106 rat myogenic stem cells/ IM [234]	↑Contract	Addition of myogenic stem cells improved function of the external anal sphincter after mechanical injury
Determined possible mechanisms by which BMSC exert their regenerative potential to injured anal sphincter	Rats/ SP	4 × 106 rat BMSC/ IV; IM [235]	↑ contract, ↑ collagen deposition	Direct, but not IV, injection of BMSC into the injured anal sphincter improved contractile function of the sphincter, increased matrix deposition in the external anal sphincter after injury
Evaluated in vivo function of the external anal sphincter after transection and repair augmented with myogenic stem cells	Rats/ SP	5 × 106 Rat myogenic stem cells/ IM [236]	↑ Anal pressure	Restoration of anal sphincter function by the transplantation of myogenic stem cells and associated trophic factors
Evaluated functional recovery of injured anal sphincter after treatment with BMSC	Rats/ SP or PNC	2 × 106 rat BMSC/ IV; IM [237]	↑ Anal pressure (SP)	BMSC treatment resulted in significant improvement in anal pressures after SP but not after PNC
Evaluated the effect of myogenic stem cells on histological properties and the volume of striated muscle of the external anal sphincter after transection and repair	Rats/ SP	3.2 × 106 rat myogenic stem cells/ IM [238]	↑ Contract	Application of myogenic stem cells to transected/ repaired anal sphincters did not alter the amount of inflammation nor the volume of striated muscle but improved contractile function
Evaluated the effect of MSC-laden hydrogel scaffold on contractile function and histomorphology of the external anal sphincter after transection injury	Rats/ SP	3.2 × 106 rat MSC (in hydrogel)/ IM [239]	↑ Ske-M, ↑ contract	A biologically compatible matrix facilitated stem cell survival, differentiation, or function leading to recovery of contractile function even after persistent disruption
Investigated whether unexpanded BMNC are effective as in vitro expanded BMSC to enhance sphincter healing after injury and primary repair	Rats/ SP	3 × 106 rat BMSC: 7.38 × 106 rat BMNC/ IM [115]	↑ Sm-M, ↑ contract	Minimally manipulated BMSC were as effective as in vitro expanded BMSC for the recovery of anal sphincter injury followed by primary sphincter repair
Assess the effects of co-application of laser and ASC on anal sphincter recovery after injury	Rabbits/ SP	2 × 106 human ASC/ IM [80]	↑ Anal pressure, ↑ Ske-M, ↑ Vas	Combination of laser and ASC were more effective than either treatment alone for promoting myogenesis, angiogenesis, and functional recovery
Lower esophageal sphincter				
Investigated whether transplantation of MDC into gastrointestinal sphincters improve their function	Rats/ Dogs	0.2 × 106 rat MDC; 4 × 106 dog MDC/ IM [106]	Rats: ↑ contract Dogs: ↑ LES pressure, ↓ acid reflux	MDC survived and integrated into gastrointestinal smooth muscle and augmented their contractile response in two LES injury animal models
Determined the role of autologous BMSC in the regeneration of the LES after surgery	Rats with esophagogastric myotomy	1.5 × 106 rat BMSC/ IM [116]	↑ Sm-M, ↑ contract	Use of BMSC improved sphincter regeneration of LES and control gastro-esophageal reflux after surgery

*ASC* adipose-derived stem cells, *BMNC* bone marrow mononuclear cells, *BMSC* bone marrow-derived mesenchymal stem cells, *contract* contractility, *IM* intramuscular, *IV* intravenous, *LES* lower esophageal sphincter, *LPP* leak point pressure, *M* myogenesis, *MDC* muscle derived cells, *MSC*: myogenic stem cells, *Peri-N* peripheral neurogenesis, *PNC* pudendal nerve crush, *Ske-M* skeletal muscle myogenesis, *Sm-M* smooth muscle myogenesis, *SP* sphincterotomy, *UCMSC* umbilical cord matrix stem cells, *UPP* urethral pressure profile, *Vas* vascularization, *VD* vaginal dilation, ↑: increased; ↓: decreased

Isolation and purification processes of MSC from the tissues are complex and critical endeavor. To confirm the stemness, isolated cells are usually tested for osteogenic and adipogenic capacity with Alizarin Red S and Oil Red O staining after appropriate cultivation; flow cytometry analysis is performed to examine the expression of mesenchymal makers (CD44, CD73, CD90, CD105, CD106) [113–116]. In addition, MSC are a heterogeneous mixture of cells, and their relatively low purity limits further clinical applications. A more homogeneous population of MSC would be more likely to exhibit consistent properties and behaviors, making it easier to control the differentiation process and generate a population of target cells with the desired properties and functions. In contrast, if the MSC are highly heterogeneous, it may be more difficult to control the differentiation process and achieve a consistent population of target cells. In addition, it may be more difficult to identify the subset of MSC with the best characteristics for differentiation into the desired target cells, which could negatively impact the efficiency and effectiveness of the differentiation process. Therefore, reducing the heterogeneity of MSC through selection or manipulation may be a useful strategy to enhance the ability of the stem cells to differentiate into specific target cells, which may be beneficial for tissue regeneration and other applications. Several methods have been proposed to purify MSC and improve transplantation efficiency. The membrane filtration method showed that the primary cell solution was permeated through membranes, and then the culture medium was permeated to detach the ASC into the culture medium. The isolated ASC showed a superior capacity for osteogenic differentiation [117]. The membrane migration method combined the membrane filtration method and the culture method. The primary cell solution was permeated through membranes, and the membranes were incubated in cell culture medium, and then the ASC migrated out of the membranes which showed high purity and pluripotency [118, 119]. Magnetically activated cell sorting isolated MSC depending on their surface antigens, such as CD90, CD146 and CD271 [120–122]. In addition, a thermoresponsive cationic block copolymer brush-grafted bead-packed column was developed to achieve ASC separation by altering the temperature without cell surface modification and cellular activity reduction [123, 124]. With the development of single-cell RNA sequencing, MSC subpopulations have been identified, indicating their respective distinct functions [125, 126]. Future research will aim to improve the purification of MSC to provide more specific stem cells at the cell subset level for the cell-based therapy. Immunohistochemical staining of specific makers of muscle, nerve and NMJ (Table 1) was performed to evaluate the effectiveness of stem cell therapy [76, 114]. However, the dynamic changes in differentiation of stem cell after transplantation in the recipient area have not been well controlled. Although green fluorescence protein (GFP) or PKH26 were used to label or track the fate of stem cell in recipient tissues, the physiology functional change of stem cell with time needs more investigations in the future.

### Clinical trial data and challenges

In order to bypass graft rejection, the use of autologous cells from the patient is the ideal treatment scenario for functional restoration of the damaged sphincter muscle. Clinicals studies using stem cell therapy for sphincter dysfunctions have been previously summarized as part of several recent reviews [127–129]. Garcia-Arranz et al. evaluated the safety and feasibility of ADSC for the treatment of male or female SUI. Urinary incontinence was significantly improved in 3 out of 8 men and 5 out of 10 women, with no adverse effects reported in any patient. Clinical improvement was maintained for more than 12 to 15 months of follow-up [130]. A recent randomized clinical trial compared the effects of autologous adult mucosa stem cell therapy with the mini-sling procedure for female SUI. Results at 6 and 26 months of follow-up showed that periurethral injection of autologous adult mucosa stem cells was not inferior to the mini-sling procedure, while the stem cell group had a shorter procedure time and hospital stay, as well as fewer complications [131]. In another randomized clinical trial, Boyer et al. reported on the efficacy and safety of MDC injection for the treatment of FI. The results showed a greater than 30% reduction in the Cleveland Clinic Incontinence (CCI) score in 58% of patients in the MDC group compared to 8% in the placebo group at 12 months post-treatment, with excellent tolerability and safety [132]. A systematic review including 11 clinical studies found that stem cell therapy for SUI and FI was a safe procedure with few mild adverse side effects [133]. However, the number of patients was limited. The outcome measures and time points were very heterogeneous. Larger targeted studies with control arms needed to be performed to give clear evidence for the beneficial impact of stem cell therapy. Another systematic review analyzed published data on the clinical therapeutic benefit and safety of urethral injections of autologous stem cells for the treatment of SUI [134]. These clinical studies showed encouraging results with minimal side-effects and complications. However, long term safety and efficacy data still needed to be investigated because the mean follow-up was less than 12 months in the available studies. Another challenge identified in this study was the relatively short life span of stem cells after transplantation. Indeed, the low survival rate of cells transplanted into the damaged area has long been a major challenge in the translation of stem cell therapy.

Improving the survival rate of cell transplantation is a key challenge in the field of regenerative medicine and cell therapy. The success of cell transplantation depends on several factors, including the type of cells used, the method of transplantation, and the environment in which the cells are transplanted. Here are some of the strategies that have been used or proposed to improve the survival rate of cell transplantation: i), Cell selection: Selecting the right type of cells for transplantation is critical for success. Stem cells, for example, have a greater potential for survival and differentiation than fully differentiated cells. However, stem cells cultured in vitro for long periods of time generally show low survival and tissue engraftment, reduced paracrine effects, and poor homing and differentiation rates [58]. This may be partly due to over expansion of cells during in vitro culture, which leads to senescence and loss of potency; ii), Transplantation method: The method of transplantation can affect the survival rate of cells. Transplanting cells in a hydrogel or scaffold can provide a supportive environment that increases cell survival and promotes tissue regeneration [135, 136]; iii), Microenvironment: The microenvironment of the transplanted cells is important for their survival. Cells need to be in an environment that is conducive to their growth and survival, with adequate oxygen and nutrients. When cells are isolated from the tissue, they lose their original microenvironment resulting in decreased viability and DNA instability after long-term culture [137, 138]; iv), Delivery of growth factors: Supplementing the transplantation with growth factors or other biological molecules can help improve cell survival. Growth factors can help promote cell survival, growth, and differentiation [139–141]; v), Immune response: The immune response can also affect the survival of transplanted cells [142]. Strategies to reduce the immune response and prevent cell rejection include the use of immune-suppressive drugs, the use of cells derived from the same individual as the transplant recipient, and encapsulation of cells in hydrogels or other materials [143, 144]. Further research is needed to fully understand the complex mechanisms involved in cell survival and to develop new and more effective strategies to improve the survival rate of cell transplantation in the treatment of sphincter dysfunction.

## Growth factor therapy

### Growth factors in sphincter dysfunction

Growth factors are signaling molecules that play a critical role in regulating cellular processes such as proliferation, differentiation, and survival. In the context of sphincter dysfunction, growth factors have been investigated as a potential therapy to improve sphincter function and promote tissue regeneration. Several growth factors have been studied for their potential role in sphincter dysfunction, including:

#### Platelet-derived growth factor (PDGF)

PDGF is a potent mitogenic and angiogenic factor that promotes cell proliferation and survival. It has been shown to regulate the proliferation, migration, and differentiation of mesenchymal cells and to promote muscle and nerve regeneration [145–149], making it a potential therapeutic option for sphincter dysfunction.

#### Transforming growth factor-β (TGF-β)

TGF-β is a growth factor that regulates cell proliferation, differentiation, and extracellular matrix production. It has been shown to promote the differentiation of mesenchymal stem cells into smooth muscle cells and to stimulate smooth muscle cell proliferation [150–154], making it a potential therapeutic option for sphincter dysfunction.

#### Fibroblast growth factor (FGF)

FGFs are a family of growth factors that play a role in cellular proliferation, differentiation, and angiogenesis. They have been shown to enhance satellite cell proliferation in vitro [155, 156] and to promote peripheral nerve regeneration after injury [157–159], making them a potential therapeutic option for sphincter dysfunction.

#### Vascular endothelial growth factor 

(VEGF): VEGF is a known promoter of angiogenesis [160]. The vasculature serves as a conduit for nutrients and oxygen and performs perfusion-independent functions as an essential component of the stem cell niche for many adult stem cells [161, 162]. Therefore, the application of VEGF to the stem cell niche may rejuvenate the microenvironment resulting in modulation of stem cell behavior during tissue regeneration [163, 164]. VEGF has also been shown to stimulate the proliferation and survival of smooth muscle cells [165, 166], and to exert provide neurotrophic and neuroprotective effects in the peripheral nervous system [167]. This makes it a potential therapeutic option for sphincter dysfunction.

#### Neurotrophic Factors (NF)

 The most well studied neurotrophic family members include brain-derived neurotrophic factor (BDNF), nerve growth factor (NGF), neurotrophin-3 (NT-3), and neurotrophin-4/5 (NT-4/5) [168]. Increasing evidence suggests that NF not only support neuronal survival, growth, and damage repair but are also involved in the development and differentiation of myoblasts and muscle fibers [169–173]. These studies highlight the therapeutic potential of NF for sphincter dysfunction.

#### Insulin-Like Growth Factor 1

(IGF-1): IGF-1 is a growth factor that is similar in structure to insulin. As an important anabolic growth factor expressed in muscle and nerve tissue, IGF-1 has been shown to promote neuronal survival, axon growth, and muscle growth [174–177], making it a potential therapeutic option for sphincter dysfunction.

It's important to note that these growth factors are still being studied for their potential role in sphincter dysfunction, and more research is needed to determine the optimal combination, dosage, duration, and frequency of growth factors for therapeutic use. In addition, the use of growth factors for sphincter dysfunction is still in the early stages of development, and more studies are needed to determine the safety and efficacy of this approach.

Overall, the mechanisms of growth factor therapy can be summarized as follows. First, growth factors directly participate in tissue repair and regeneration by activating intrinsic growth programs of damaged neuromuscular tissues through the regulation of intracellular transcription factors. Second, growth factors enhance the therapeutic efficacy of stem cells. They interact with cell-specific receptors to initiate extracellular and intracellular signaling cascades responsible for the physiological function of stem cells. Third, application of growth factors to the stem cell niche may rejuvenate the microenvironment, resulting in modulation of stem cell behavior during tissue regeneration. Therefore, focusing on how to maximize the benefits of growth factors and how to maintain the bioactivity of growth factors may guide us in the search for a promising therapeutic strategy for the treatment of sphincter dysfunction.

### Methods of controlled release

#### Genetic modification

Gene therapy is the transfer or modification of genetic material to a patient to treat a disease [178]. In addition to correcting an existing abnormality to elicit a therapeutic effect, gene therapy can also be a tool to deliver a gene of interest into cells in order to restore or increase expression of a protein [179]. Various non-viral and viral vectors have been used for gene delivery in order to modify the genome of the target cell. Non-viral systems generally utilize physical and chemical systems. Physical methods of delivering the desired gene of interest include the use of naked DNA, the gene gun, electroporation, hydrodynamic or ultrasound transfection, and magnetofection, and chemical methods include the use of cationic liposomes and cationic polymers [180, 181]. The benefits of the non-viral methods include safety, cost-effectiveness, low host immunogenicity, and an unlimited transgene size. However, the efficiency of these methods is limited.

Viral methods of gene delivery, by contrast, is a very efficient method of introducing exogenous DNA into cells. Viral vectors that have been used for this purpose include retrovirus, adenovirus, adeno-associated virus (AAV), herpes simplex virus (HSV), lentivirus, and poxvirus [178, 180]. In our previous work, we genetically modified stem cells to express growth factors such as VEGF [56], FGF2 [182] or pigment epithelium-derived factor (PEDF) [183] via lentiviral transduction for in vivo tissue repair in the urinary tract system. The exogenous growth factors were continually secreted from the transfected stem cells over 30 days [56]. In a rat model of erectile dysfunction, the application of genetically modified ASC/USC significantly improved erectile function, compared to stem cell implantation alone [56, 182, 183].

All viral vector genomes have been modified so that they are reproduction incompetent to ensure their safety. However, issues associated with viral use such as complicated processes of vector production, limitations in the size of transgenic DNA, potential deleterious immune responses, and in rare cases, loss of life remain [178–180, 184]. Better genome editing techniques (i.e., CRISPR/Cas9 system) have been developing at a rapid pace, which enables targeted genome modifications in cells and reduces the risks of genotoxicity in viral vector-based gene therapy. However, the off-target activity and the induction of DNA modifications at unintended sites currently limits their utility in gene therapy [185, 186].

#### Biomaterial-based delivery systems

In recent years, biomaterial-based delivery systems have become an increasingly popular method for use with cell transplantations. Multiple properties of biomaterials, including crystallinity, glass transition temperature, solubility, molecular weight, dispersity, surface charge, degradation rates, diffusion rates, and dissolution rates are crucial factors that can influence the successful delivery of growth factors [187]. To achieve acceptable growth factor release kinetics, the biomaterials used should have good biocompatibility, loading efficiency, molding properties, the ability to protect growth factors from decomposition, and the ability for continuous growth factor release [68, 70].

Various structures, including microspheres, nanoparticles, liposomes, extracellular vesicles, etc., have been studied as carriers in delivery systems. Among these delivery devices, alginate hydrogel microspheres, often used with microbeads, is widely studied for its biocompatibility, biodegradability, tunability in mechanical property, and sustained release ability [188–191]. In our previous work, we developed injectable alginate microbeads encapsulating three growth factors (i.e., VEGF, IGF and FGF-1). To control the release of growth factors from the microbeads, a semi-permeable membrane of poly-L-ornithine (PLO) was used to decrease the porosity of the alginate microbeads from 600 kDa to about 70–80 kDa. The release kinetics of VEGF, IGF, and FGF-1 from the alginate microbeads showed as a stable and continuous curve for 30 days in vitro. The bioactivity of growth factors was preserved as evidenced by the angiogenic differentiation of USC in the presence of VEGF embedded microbeads [55]. To enhance the retention of microbeads at a local site, we developed microbeads coated with multiple layers of hydrogel. We used gelatin as the outer layer of the microbeads, as the hydrophilicity of gelatin aids in the binding of microspheres to the host tissue [192]. The results showed better retention of alginate-PLO-gelatin (A-PLO-G) microbeads around urethral tissue after periurethral injection, and A-PLO-G microbeads loaded with IGF-1 significantly promoted skeletal regeneration and angiogenesis in the SUI rat model [193].

As growth factors are usually unable to efficiently bind to biomaterials, a bridge for covalent crosslinking is required. Heparin, a highly sulfated anionic glycosaminoglycan, contains a growth factor binding domain that allows it to bind growth factors with high affinity and control the release while protecting them from thermal denaturation and enzymatic degradation. This interaction occurs partly due to shape recognition, but mainly due to the electrostatic attraction between N- and O-sulfated residues of heparin and the lysine/arginine residues of growth factors [194–196]. Zhao et al. developed tissue-engineered nerves based on a VEGF-heparin sustained controlled release system to promote peripheral nerve defect regeneration and repair in rats [197]. The results showed early vascularization and restored blood supply in the nerve graft area. Our previous work showed that in vivo delivery of growth factors from a hyaluronic-heparin hydrogel maintained the bioactivity of growth factors, as well as increased the resulting myogenic and angiogenic responses over 28 days, through long-term localized release [58].

Tissue-specific ECM, which provides an in vitro microenvironment that is similar to the in vivo environment, is able to promote cell growth and retention of cellular phenotypes [198]. However, growth factors in the ECM are rapidly degraded or washed-out during culture in vitro. To achieve controlled release of bioactive growth factors from ECM, heparin linked with hydrogel was used. Our studies demonstrated that tissue-specific ECM combined with heparin conjugated hyaluronic acid hydrogel was capable of binding growth factors for sustained release and maintenance of a constant level of growth factor-cell signaling in the local environment [199, 200]. This resulted in better outcomes in cell viability, proliferation, and differentiation than hydrogel or ECM application alone.

### Pre-clinical data in sphincter dysfunction

Multiple preclinical studies have investigated the use of growth factors to improve sphincter dysfunction (Table 3). These investigations indicated that the use of growth factors, either on their own or in conjunction with controlled release methods, have the potential to improve sphincter function. Although evidence from preclinical studies supported that growth factor therapy serve in multiple capacities of neuromuscular repair and regeneration, there remains a big gap between injury models and clinical scenarios. The use of growth factors in delivery system still presents major challenges due to their unstable bioactivity and uncertain safety. Up till now, few clinical data about growth factor therapy alone for sphincter dysfunctions has been published. VM202 (Engensis^®^), developed by Helixmith, is a plasmid DNA product designed to produce two isoforms of hepatocyte growth factor (HGF). VM202 has been studied in experimental models and clinical trials for therapeutic benefit in several diseases, including critical limb ischemia, myocardial infarction, amyotrophic lateral sclerosis, and painful diabetic peripheral neuropathy [201–209]. The mechanism of action may be attributed to the restoration of damaged nerves and blood vessels via the neurotrophic and angiogenic activities of HGF [210–212]. This provides a glimpse into the potential of growth factor therapy in neuromuscular repair and regeneration.

**Table 3:** Selected pre-clinical studies of growth factors therapy for sphincter dysfunctions

Goal of studies	Animal model	Growth factors/ deliver methods	Main outcomes	Conclusions
Urethral sphincter				
Determined the effects of controlled release of IGF-1 from A-PLO-G microbeads on external urethral sphincter tissue regeneration in a rat model of SUI	Rats/ VD	1 × 104 IGF-1-A-PLO-G microbeads/ Periurethral injection [193]	↑ LPP, ↑ Ske-M, ↑ Vas	Periurethral administration of IGF-1-A-PLO-G microbeads facilitated recovery from SUI by promoting skeletal myogenesis and revascularization
Evaluated the effects of sustained release bFGF injection in rat urethra	Rats/ urethra denervated by Botox	50 and 200 μg bFGF in gelatin hydrogels/ Periurethral injection [240]	↑ LPP, ↑ Ske-M, ↑ Sm-M, ↑ Vas	Sustained release bFGF injection in the chemically denervated urethral sphincter facilitated regeneration of the urethral muscles and improved sphincteric contractility
Investigated the ability of neurotrophin therapy to facilitate recovery of the neuromuscular continence mechanism	Rats/ PNC + VD	2 μg BDNF/ day/ Subcutaneous miniature-osmotic pumps [12]	↑ LPP, ↑ Ske-M, ↑ Peri-N	Continuous targeted neurotrophin therapy accelerated continence recovery after simulated childbirth injury
Investigated the growth factor-immobilized porous beads as an injectable urethral bulking agent	Rats/ sciatic nerve transection	20 μl bFGF or VEGF immobilized porous beads/ Periurethral injections [241]	↑ LPP, ↑ Sm-M, ↑ contract	Growth factor-immobilized porous beads were a good candidate as an injectable bioactive bulking agent
Investigated the effect of dual growth factor-loaded in situ gel-forming bulking agent for the treatment of SUI	Rats/ sciatic nerve transection	20 μl NGF and/ or bFGF loaded bulking agents/ Periurethral injections [242]	↑ LPP, ↑ Sm-M, ↑ Peri-N, ↑ contract	NGF/ bFGF-loaded in situ gel-forming bulking agent might be a promising injectable bioactive system for the treatment for SUI
Assessed whether HGF combined with IGF-1 promote the activation, proliferation, and differentiation of satellite cells	Rats	10 μl IGF-1 (50 μg/ ml) and 10 μl HGF (20, 50, 100 μg/ ml)/ Periurethral injections [243]	↑ Ske-M	Synergistic effect of HGF and IGF-1 injection on skeletal muscle regeneration
Anal sphincter				
Investigated effects of IGF-I gene therapy on the injured rat pudendal nerve	Rats/ PNC	25 μl recombinant human IGF-1 plasmid (75 mg DNA dose)/ IM [244]	↑ Peri-N	IGF-I gene therapy might improve the distal recovery of structure and function of pudendal nerve
Compared several growth factors to optimize the growth and survival of a bioengineered internal anal sphincter	Mice	10 μg of FGF-2, 2 μg of VEGF or 2 μg of PDGF/ Subcutaneous micro-osmotic pump [245]	↑ Sm-M, ↑ Vas	All growth factors displayed encouraging performance, particularly PDGF-supplemented implants after in vivo implantation

*A-PLO-G* alginate-poly-L-ornithine-gelatine, *BDNF* brain-derived neurotrophic factor, *bFGF* basic fibroblast growth factor, *Botox* botulinum-A toxin, *HGF* hepatocyte growth factor, *IGF-1* insulin-like growth factors 1, *IM* intramuscular, *LPP* leak point pressure, *NGF* nerve growth factor, *PDGF* platelet-derived growth factor, *Peri-N* peripheral neurogenesis, *PNC* pudendal nerve crush, *Ske-M* skeletal myogenesis, *Sm-M* smooth muscle myogenesis, *Vas* vascularization, *VD* vaginal dilation, *VEGF* vascular endothelial growth factor; ↑: increased

## Combination therapy of stem cells and controlled released of growth factors

### Neuromuscular regeneration using dual therapy

In a controlled release system, stem cells, growth factors, and biomaterial carriers come together to form a geometric unit. Growth factors released from biodegradable microbeads or gels can be locally applied to improve cell retention, viability, differentiation, and paracrine signaling of the grafted stem cells. In addition, they can stimulate and recruit endogenous stem cells to facilitate tissue injury repair and regeneration. Stem cell therapy combined with a controlled release of growth factors has the potential to result in increased neuromuscular regeneration by increasing tissue and graft angiogenesis, myogenesis, and innervation, compared to treatment alone.

### Pre-clinical data in sphincter dysfunction

To date, there are few combination therapies for anal sphincter and LES dysfunction, and few pre-clinical studies that have shown only modest progress in the treatment of urethral sphincter dysfunction [55, 57–59]. The standard treatment of SUI, for example, requires restoration of a functional urethral sphincter which requires a vascularized urethral submucosal layer, striated and smooth muscle components, and effective innervation to maintain urinary continence [213]. Zhao et al. reported that periurethral injection of autologous ASC with controlled-release nerve growth factor (NGF) enhanced the therapeutic efficacy of ASC in a rat model of SUI [59]. In this study, NGFs encapsulated in PLGA microspheres were demonstrated to improve ASC viability in vitro and in vivo. The combination of ASC and PLGA loaded with NGF significantly improved bladder capacities as evidenced by enhanced abdominal leak point pressure and retrograde urethral perfusion pressure. Histologic evaluation of the urethra showed increased muscle and neuronal density after ASC and PLGA-NGF treatment.

In our previous work, we studied the combination therapy of un-differentiated USC with controlled release of growth factors from a heparin-hyaluronic acid (hp-HA) gel via subcutaneous injection in mice [58]. The growth factor cocktail released from the hp-HA gel containing IGF1, HGF, PDGF, NGF, FGF and VEGF increased USC retention at the site of injection and enhanced the recruitment of resident cells into the graft. Additionally, the myogenic differentiation potential of USC was increased in the combination therapy group, as evidenced by increased expression of early myogenic markers (Desmin, Myf-5 and Myo-D) in the graft. Moreover, increased vasculature (marked by CD31, vWF) and nerves (marked by neurofilament) in the graft were detected in the combination group as compared to the growth factor alone treated group. Lastly, the combined use of growth factors and cells required 1/3 the dose than that of the growth factor alone. The use of decreased doses of growth factors may reduce the risk of side effects due to supra-physiological doses when used alone [58]. In another study, we used USCs combined with growth factors embedded in alginate microspheres. The growth factors included IGF1, HGF, PDGF, NGF, FGF and VEGF. After subcutaneous injection into mice, the histologic analysis showed enhanced myogenesis, angiogenesis, and peripheral nerve formation in the graft [55].

### Clinical trial data

Although there are currently no clinical trials of combination therapy for sphincter dysfunction, the combination therapy has shown positive results in the treatment of other diseases. NurOwn^®^, developed by BrainStorm Cell Therapeutics, is a therapy that combines the administration of MSC and NF to treat patients with ALS. Autologous MSC are harvested from the bone marrow and then induced to secrete NF. These MSC-NF cells have the potential to protect motor neuron survival and promote neuromuscular junction reinnervation. Clinical results suggest that the MSC-NF cells transplantation is safe and well tolerated, with improvements in ALS Functional Rating Scale-Revised score and forced vital capacity [214–216]. A randomized controlled clinical trial was conducted to evaluate the efficacy of MSC cultured on beta-tricalcium phosphate in combination with recombinant human PDGF-BB in the treatment of human infrabony defects. The results showed that clinical parameters such as pocket depth, clinical attachment level, and radiographic bone volume tended to improve after combination therapy at 6-month follow-up [217]. Another clinical trial showed that combination therapy with stem cells and activated platelet-rich plasma growth factor concentrate was promising for cartilage regeneration, as evidenced by improved MRI data in 45 of 48 patients with avascular necrosis of the femoral head [218]. The information gained from these combination studies is expected to lead to the development of new neuromuscular regeneration therapies.

### Animal models of sphincter dysfunction

As shown in Table 2 and Table 3, various animal models of sphincter disorders can be useful for studying cell therapy or growth factor therapy for these conditions, but they also have advantages and disadvantages. Advantages include: (i), Relevance: animal models can provide a relevant and physiologically accurate representation of the human condition, making them useful for testing cell therapy or growth factor therapy for sphincter disorders; (ii), Control: in animal models, it is possible to control various environmental and experimental factors, such as diet, hormonal status, and disease state, which may be difficult to control in human studies. This can provide a more controlled and standardized experimental setting for studying the efficacy of cell therapy or growth factor therapy for sphincter disorders; (iii), Cost: animal models can often be less expensive than human studies, especially if they involve complex procedures or expensive drugs or devices. Disadvantages include: (i), Species differences: there may be differences between humans and the animal models used, such as differences in anatomy, physiology, or disease mechanisms, which may limit the generalizability of the results to humans; (ii), Ethical considerations: the use of animal models for research may raise ethical concerns, particularly when the animals are subjected to procedures that cause pain or distress; (iii), Limited representation: animal models may not accurately represent all aspects of the human condition, especially if the sphincter disorder being studied is rare or has complex underlying mechanisms; (iv), Interpretation of results: interpreting the results of animal studies can be challenging, especially if the results are not directly comparable to human studies. This may limit the ability to draw meaningful conclusions about the potential efficacy of cell therapy or growth factor therapy in humans; (v), Limited cell transplantation methods: The methods available for cell transplantation in animal models may be limited and may not accurately reflect the methods that would be used in humans.

In the preclinical studies, several animal models have been developed to injure different aspects of the sphincter function. For example, SUI animal models can be induced by vaginal dilation (VD), pudendal nerve injury or dual injury, which simulates the vaginal birth trauma [219, 220]. However, these animal models have several unavoidable limitations. First, sphincter dysfunction is a complex and usually multifactorial process, sometimes involving a combination of denervation, muscle degeneration, atrophy and fibrosis [221]. It is almost impossible for an in vivo model to integrate all the pathophysiological factors that fully simulate the human situation. Second, some models are unique in their reversibility feature. This means that the sphincter dysfunction resolves spontaneously, making it difficult to observe the long-term effects of cell-based therapy. For example, the VD model has a limited duration of the sphincter functional and structural sphincter defects, with a recovery of leak point pressure within 10 days, and up to 6 weeks [221–223]. Third, the assessment of sphincter dysfunction relies heavily on subjective symptoms reported by patients via questionnaires, such as urine leakage on cough in SUI patients and frequency of heartburn in GERD patients. This in not possible in animal models. Surrogate outcome measures in animal models may not accurately reflect the effect of therapy.

Overall, animal models of sphincter disorders can be valuable tools for studying cell therapy or growth factor therapy for these conditions. However, it is important to carefully consider the advantages and disadvantages of these models when designing and interpreting research studies, and to validate the results in human studies before moving to clinical applications.

## Conclusion and future prospects

Sphincter dysfunction is a consequence of neuromuscular impairment that occurs with aging, trauma and inflammation in various lumen tissues. Undoubtedly, stem cell-based therapies aimed at restoration of neuromuscular function have emerged as an attractive approach in regenerative medicine. Compared to the use of stem cells alone, combination therapy of stem cells with controlled release of growth factors significantly enhances cell viability of the implanted cells by increasing paracrine effect and recruiting the host stem cells to participate to the internal tissue regeneration. Preclinical studies displayed that combination therapies are promising and safe in short-term experiments. However, its long-term efficacy and safety remain unknown that needs to be further investigated. If it is successful, stem cell therapy with consistently delivered growth factors can be used in the other sphincter disorders (i.e. vesicoureteral reflux and sphincter of oddi dysfunction) and muscular dystrophy.

The biomaterial-based delivery system provides an effective controlled-release vehicle for multiple growth factors to improve stem cell survival and function. It should also be noted that stem cell adhesion and differentiation can be influenced by the stiffness of the hydrogel used [224–226] suggesting that the effects of the mechanical properties of the hydrogel on stem cells needs to be considered. The fate of cells grown on various hydrogels needs to be investigated while taking into consideration the biophysical cues from the dual microenvironment of the hydrogel and the native environment of the recipient after transplantation. Additionally, neuromuscular regeneration is a dynamic process that is regulated by numerous growth factors that coordinate with each other to regulate this process [141].

To achieve ideal functional repair of damaged muscles and nerves that mimic the body's physiological process, the delivery of multiple growth factors using various carrier biomaterials in specific spatial and temporal ways needs to be considered. Recently, extracellular vesicles have emerged as promising drug carriers. Advantages of exosomes as carriers include high biocompatibility, enhanced stability, and limited immunogenicity [227, 228]. Wang et al. loaded recombinant human NGF protein and mRNA into exosomes. The engineered exosome showed long-term stability, effectively delivered NGF into the ischemic region and reduced ischemic injury in mice [229]. In another study by Li et al., delivery of VEGFC, via engineered exosomes in a sodium alginate hydrogel, improved lymphedema in mice [230]. Therefore, a vesicle-based delivery strategy may also hold promise as an advanced platform for assisting stem cell therapy.

## Data Availability

Not applicable.
